# Quality of life and financial toxicity of hematopoietic stem cell transplant recipients in COVID-19

**DOI:** 10.1590/1518-8345.6688.3996

**Published:** 2023-09-18

**Authors:** Natália Naome Oshiro, Luciana de Alcantara Nogueira, Yasmin Hiorrana dos Santos, Paulo Ricardo Bittencourt Guimarães, Luciana Puchalski Kalinke

**Affiliations:** 1 Universidade Federal do Paraná, Departamento de Enfermagem, Curitiba, PR, Brasil.; 2 Universidade Federal do Paraná, Departamento de Estatística, Curitiba, PR, Brasil.

**Keywords:** Quality of Life, Hematopoietic Stem Cell Transplantation, Bone Marrow Transplantation, Financial Stress, Financial Toxicity, COVID-19, Calidad de Vida, Trasplante de Células Madre Hematopoyéticas, Trasplante de Médula Ósea, Estrés Financiero, Toxicidad Financiera, COVID-19, Qualidade de Vida, Transplante de Células-Tronco Hematopoéticas, Transplante de Medula Óssea, Estresse Financeiro, Toxicidade Financeira, COVID-19

## Abstract

**Objective:**

to evaluate and correlate the quality of life and financial toxicity of adult patients undergoing hematopoietic stem cell transplantation during the COVID-19 pandemic.

**Method:**

observational, analytical study, carried out with 35 patients in a reference hospital for transplantation in Latin America. For data collection, the Functional Assessment Cancer Therapy Bone Marrow Transplantation and COmprehensive Score for Financial Toxicity questionnaires were used. Spearman and Mann-Whitney correlation tests were used for data analysis.

**Results:**

general quality of life during COVID-19 had a low score (67.09/108) with greater impairment in functional well-being (14.47/28), social well-being (16.76/28) and additional concerns (23.41/40). The means of the allogeneic group were lower than those of the autologous group in all domains, showing a significant difference in relation to additional concerns (p=0.01) and in the treatment evaluation index (p=0.04). Financial toxicity was considered to have a slight impact (22.11/44). There was a relationship, albeit not significant, between quality of life and financial toxicity (p=0.051).

**Conclusion:**

the quality of life of the sample was low; there is a correlation between quality of life and financial toxicity, although not significant. The higher the financial toxicity, the lower the quality of life.

## Abstract (es)

**Objetivo:**

evaluar y correlacionar la calidad de vida y la toxicidad financiera de pacientes adultos sometidos a trasplante de células madre hematopoyéticas durante el período de la pandemia de COVID-19.

**Método:**

estudio observacional, analítico, realizado con 35 pacientes en un hospital de referencia para trasplante en Latinoamérica. Para la recolección de datos, se utilizaron los cuestionarios Functional Assessment Cancer Therapy Bone Marrow Transplantation y el COmprehensive Score for financial Toxicity. Para el análisis de los datos se utilizaron las pruebas de correlación de Spearman y Mann-Whitney.

**Resultados:**

la calidad de vida general durante la COVID-19 mostró un puntaje bajo (67,09/108) con mayor deterioro en el bienestar funcional (14,47/28), bienestar social (16,76/28) y preocupaciones adicionales (23,41/40). Los promedios del grupo alogénico fueron inferiores a los del grupo autólogo en todos los dominios, presentando diferencia significativa en relación a preocupaciones adicionales (p=0,01) y en el índice de evaluación del tratamiento (p=0,04). Se consideró que la toxicidad financiera tenía un impacto leve (22.11/44). Se observó una relación, aunque no significativa, entre la calidad de vida y la toxicidad financiera (p=0,051).

**Conclusión:**

la calidad de vida de la muestra fue baja; existe una correlación entre la calidad de vida y la toxicidad financiera, aunque no significativa. Cuanto mayor es la toxicidad financiera, menor es la calidad de vida.

## Abstract (pt)

**Objetivo:**

avaliar e correlacionar a qualidade de vida e a toxicidade financeira dos pacientes adultos submetidos ao transplante de células-tronco hematopoéticas no período da pandemia de COVID-19.

**Método:**

estudo observacional, analítico, realizado com 35 pacientes em um hospital de referência para o transplante na América Latina. Para coleta de dados, utilizaram-se os questionários Functional Assessment Cancer Therapy Bone Marrow Transplantation e COmprehensive Score for financial Toxicity. Na análise dos dados empregaram-se os testes de correlação de Spearman e Mann-Whitney.

**Resultados:**

a qualidade de vida geral, durante a COVID-19, apresentou baixo escore (67,09/108), com maior comprometimento nas funções bem-estar funcional (14,47/28), social (16,76/28) e preocupações adicionais (23,41/40). As médias do grupo alogênico foram inferiores às do autólogo em todos os domínios, apresentando diferença significativa em relação às preocupações adicionais (p=0,01) e ao índice de avaliação do tratamento (p=0,04). A toxicidade financeira foi considerada de impacto leve (22,11/44). Observou-se relação, ainda que não significativa, entre a qualidade de vida e a toxicidade financeira (p=0,051).

**Conclusão:**

a qualidade de vida da amostra foi baixa, logo há uma correlação entre qualidade de vida e a toxicidade financeira, embora não significativa. Quanto maior a toxicidade financeira, menor a qualidade de vida.

**Highlights:**

**(1)** Financial toxicity during COVID-19 was considered mild.

**(2)** Bone marrow transplant recipients had a worsening in their overall quality of life.

**(3)** The greater the financial toxicity, the worse the quality of life of transplant recipients.

## Introduction

Among the various therapeutic options for patients with cancer and/or hematological diseases, hematopoietic stem cell transplantation [HSCT (in Portuguese *transplante de células-tronco hematopoéticas-TCTH)*] is an alternative treatment with potential for survival and cure ^(^ 1 ^)^. It aims to restore bone marrow and immune function and for that, it includes therapeutic regimens that use high-dose chemotherapy and/or radiotherapy, therapy with immunosuppressants, among other medications that can affect various organs and tissues ^(^ 2 ^)^. It is divided into: autologous (stem cells from the patient) and allogeneic (stem cells from another donor, whether from a related donor or not). Autologous HSCT presents faster recovery of immune function and allogeneic transplantation is associated with a higher risk of infection ^(^ 1 ^)^. 

Among the possible complications, viral infections are some of the the main causes of morbidity and mortality in populations that underwent HSCT ^(^ 2 ^)^. For this reason, the emergence of COVID-19 has become a new challenge for these patients, mainly due to the compromised immune system. The COVID-19 respiratory syndrome, caused by an RNA-beta coronavirus called SARS-CoV-2, has been classified as a Public Health Emergency of International Concern by the World Health Organization (WHO) ^(^ 3 ^)^. As of early May 2023, there were over 6.87 million documented deaths worldwide ^(^ 3 ^)^. 

During the COVID-19 pandemic, morbidity and mortality were substantially higher in HSCT recipients than in the general population ^(^ 4 ^,^ 5 ^,^ 6 ^,^ 7 ^,^ 8 ^-^ 9 ^)^. Studies indicate that patients awaiting the procedure, those undergoing allogeneic or autologous HSCT, and those more than one year after the transplant who did not receive immunosuppression, comprise a susceptible population, in which SARS-CoV-2 infection results in tragic and even fatal consequences. This is due to treatment-related toxicity, especially with regard to respiratory and infection-related complications ^(^ 10 ^,^ 11 ^,^ 12 ^-^ 13 ^)^. 

The threats of infection by the new coronavirus and the need to reorganize health services have made transplant recipients incorporate more rigid care, especially with regard to social distancing, due to the ban on visits during hospitalization ^(^ 14 ^)^. The fear of being infected by the new coronavirus, added to the feeling of loneliness and the economic crisis caused by the pandemic, increased stress levels and possibly had a negative impact on quality of life (QoL), in its different domains (physical, psychological, social, spiritual) ^(^ 14 ^-^ 15 ^)^. 

QoL was conceptualized by the WHO as “the individual’s perception of their insertion in life, in the context of the culture and value systems in which they live, in relation to their goals, expectations, standards and concerns” ^(^ 16 ^)^. It involves spiritual, physical, mental, psychological and emotional well-being, social relationships and, more recently, it has been associated with the presence of financial toxicity (FT). FT is conceptualized as the economic impact experienced by patients who have financial difficulties in meeting the expenses arising from the treatment and, therefore, do not fully adhere to the prescriptions, have increased anxiety, change in life habits and indebtedness, situations that can cause losses to QoL ^(^ 17 ^)^. 

Indications that financial issues influence the QoL of transplanted patients can be observed in the therapeutic itinerary of these patients during professional practice and in the instrument Functional Assessment Cancer Therapy– Bone Marrow Transplantation (FACT-BMT), when asking whether the cost of treatment is considered a burden. Therefore, studies that show the existence of FT among patients undergoing HSCT are relevant and innovative for nursing, as they broaden the view on care, which is sometimes centered on physiological issues.

Studies in cancer patients have described the implications of isolation and the financial risk experienced by those working-age during the COVID-19 pandemic ^(^ 18 ^-^ 19 ^)^. Social distancing, prolonged quarantine, isolation at home due to the risk of contamination, long treatment period, indisposition due to side effects, time incompatibility between consultations and work possibly increased the chances of unemployment ^(^ 15 ^)^ and impact on quality of life. The pandemic also aggravated the situations commonly described as consequences of financial toxicity: the first was the worsening of anxiety and depression and the second was non-adherence or withdrawal from treatment as a way to minimize financial losses ^(^ 20 ^)^. 

Within the context that HSCT is an aggressive treatment that impacts on the daily and professional activities of transplant recipients and that the advent of the pandemic possibly worsened both their quality of life and financial toxicity, the guiding question of this study arose: did hematopoietic stem cells have a change in quality of life and financial toxicity during the period of the COVID-19 pandemic? Thus, the objective of this study was: to evaluate and correlate the quality of life and financial toxicity of adult patients undergoing HSCT during the COVID-19 pandemic.

## Method

### Study design

Observational, cross-sectional, analytical study that followed the recommendations of Strengthening the Reporting of OBservational Studies in Epidemiology (STROBE).

### Setting

The study was carried out at the Bone Marrow Transplant Service (BMTS) of a university hospital in the city of Curitiba - state of Paraná (PR), a reference for treatment in Latin America. The outpatient sector has 12 bed-days and patients are seen in the outpatient clinic to receive immunosuppressants, antibiotics, electrolytes, hydration, transfusions of blood components, collection of laboratory tests, dressings and maintenance of central venous catheters, according to the need and clinical picture demand.

### Population and selection criteria

All patients who underwent HSCT who were undergoing outpatient care and who met the following eligibility criteria were invited to participate in the study: age equal to or greater than 18 years, who underwent autologous or allogeneic HSCT, who presented within 100 days post-transplant (D+100), conscious and able to verbalize. It is noteworthy that the 100 days after HSCT represent the end of the critical period of the transplant, when the patient remains in outpatient care. In all, 49 patients aged 18 years or older underwent transplantation during the data collection period and were able to participate in the study. Of these, 11 progressed to death before completing 100 days after HSCT and three did not attend the evaluation and data collection consultations, being considered as a loss of follow-up. After applying the eligibility criteria, 35 patients (29 allogeneic and six autologous) participated in the study.

### Data collection and period

Data collection took place between July 2021 and July 2022. It was carried out according to the scheduling of patients, who were approached individually at the nursing office and completed three questionnaires: 1) Sociodemographic and clinical questionnaire with questions about sex, age, marital status, comorbidities, previous treatments, among others; 2) Questionnaire for measuring QoL, the Functional Assessment Cancer Therapy – Bone Marrow Transplantation (FACT-BMT) – version 4.0 and 3) the COmprehensive Score for Financial Toxicity (COST) questionnaire, to assess financial toxicity. Questionnaires 2 and 3 were translated and validated for Brazil ^(^ 21 ^-^ 22 ^)^, authorized upon registration and made available by email to researchers. 

The FACT-BMT is a self-administered questionnaire prepared by the Functional Assessment of Chronic Illness Therapy (FACIT) group to assess the quality of life of patients undergoing HSCT. It consists of 50 items, divided into five domains, one of which is specific to HSCT, and four are generic and form part of the Functional Assessment of Cancer Therapy General (FACT-G) questionnaire used to measure the quality of life of patients with any type of cancer ^(^ 21 ^)^. 

The four generic domains of the FACT-BMT are: physical well-being (seven items that encompass aspects such as physical state, nausea and pain), social/family well-being (seven items about social and family relationships), functional well-being (seven items on ability to perform activities of daily living) and emotional well-being (six items that include sadness, concern about worsening and death). The specific domain of HSCT, entitled additional concerns, is composed of 23 items that address the specific effects of the treatment ^(^ 21 ^)^. 

FACT-BMT responses are arranged on a five-point Likert scale, with scores ranging from zero (not at all) to four (very much). The score is made considering the score of each domain, as follows: in the emotional well-being domain, values range from 0 to 24; in the physical well-being, social and family well-being and functional well-being domains, they range from 0 to 28 points each. Additional concerns, related to the specific domain of HSCT, are scored from 0 to 40, and it is noteworthy that in version 4.0, with 23 questions, the score is limited to 10 items. The score of the treatment outcome assessment index (Trial Outcome Index-TOI) occurs from the sum of the physical well-being/functional well-being/additional concerns domains, which can vary from 0 to 96 ^(^ 21 ^)^. 

The FACT-G score ranges from 0 to 108 with the sum of the subscales physical well-being/social and family well-being/emotional well-being/functional well-being; for the FACT-BMT, the questionnaire score ranges from 0 to 148 and is obtained from the sum of the scores for the domains physical well-being, social and family well-being, emotional well-being, functional well-being and additional concerns. Negatively constructed questions that have a maximum value of four (very much) have a reverse score, that is, the score is transformed to zero. In the final score, higher scores represented better quality of life ^(^ 21 ^)^. 

The COST is an instrument also developed by the FACIT group, which measures financial toxicity as a single construct. It is a one-dimensional questionnaire, containing 12 items with answers on a five-point Likert scale, which can range from zero (not at all) to four (very much). The COST score ranges from zero to 44, and the higher the score, the greater the financial well-being, that is, the lower the financial toxicity ^(^ 22 ^)^. To calculate the score, question number 12 is disregarded because it is a summary item and questions number two, three, four, five, eight, nine and ten are reversed. 

For the analysis of financial toxicity, the Japanese classification was used, which classifies the impact of financial toxicity based on the score, being grade 0 - score greater than 26 (no impact); grade 1 - score between 14-25 (light impact); grade 2 - score ranging from 1-13 (moderate impact) and grade 3 refers to score 0 (high impact) ^(^ 22 ^)^. 

To calculate the FACT-BMT and COST scores, the Scoring Guideline of each questionnaire was used ^(^ 21 ^,^ 22 ^-^ 23 ^)^. 

### Data analysis

Sociodemographic and clinical data were organized in a Microsoft Office Excel ^®^ spreadsheet, analyzed descriptively and expressed as mean, absolute and relative frequency. Data obtained with FACT-BMT and COST were analyzed according to the Functional Assessment of Chronic Illness Therapy-FACIT guidelines ^(^ 23 ^)^ and expressed as mean (M) and standard deviation (SD). The relationship between the scores and domains of the instruments was performed using Spearman’s correlation coefficient. The Mann-Whitney test was applied to compare the QoL of autologous and allogeneic patient groups. For both tests, p values <0.05 were considered statistically significant. The Statistical Package for the Social Sciences (SPSS) software, version 20, was used to carry out the tests. 

### Ethical aspects

The research was approved by the Research Ethics Committee with Human Beings of the institution where the research was carried out under opinion number 4,894,397. The Informed Consent Form [ICF, (in Portuguese *Termo de Consentimento Livre e Esclarecido-TCLE*), was read, requesting authorization to complete the questionnaires and emphasizing that all information would be kept confidential. 

## Results

From July 2021 to July 2022, 49 HSCT were performed; of these, 11 died before completing 100 days and three did not return for consultations, thus 35 patients participated in the research. Of these, 82% (n=29) underwent allogeneic HSCT.

The sociodemographic characterization of transplant recipients showed that the mean age was young adults, 43 years old, 60% (n=21) were male; 68% (n=24) were married or declared a stable relationship. Regarding education, 54% (n=19) had completed high school, and 80% (n=28) declared themselves to be economically active. As for the clinical characterization, 42% (n=15) had a diagnosis of leukemia, 45% (n=16) had some comorbidity, eight (23%) had COVID-19 prior to the transplant.

Regarding the quality-of-life scores, measured by the FACT-BMT instrument, a low value was observed in the average of the general evaluation in both modalities (80.50/108 for autologous and 67.09/108 for allogeneic). In the other domains, the lowest means were: functional well-being (14.47/28), social well-being (16.76/28) and additional concerns (23.41/40) ( Table 1). 

**Table 1 -:** Functional Assessment of Cancer Therapy Bone Marrow Transplantation (FACT-BMT*) scores of patients undergoing autologous and allogeneic transplantation (n=35). Curitiba, PR, Brazil, 2021-2022

Domains Mean (DP)	Autologous (n=6)	Allogenic (n=29)	***P***
	Mean (DP)		
FACT- BMT*	110,17 (22,96)	90,51 (17,15)	0.068
Physical well-being	23,00 (3,58)	18,45 (5,75)	0.062
Social well-being	19,50 (6,25)	16,76 (4,98)	0.356
Emotional well-being	19,33 (3,93)	17,41 (4,25)	0.312
Functional well-being	18,67 (6,31)	14,47 (5,09)	0.112
Additional concerns	29,67 (5,09)	23,41 (5,42)	0.012
TOI†	71,33 (14,76)	56,33 (13,95)	0.044
FACT-G‡	80,50 (18,06)	67,09 (13,08)	0.068

Note: Mann Whitney test

* FACT-BMT = Functional Assessment of Cancer Therapy Bone Marrow Transplantation;

† TOI = Trial Outcome Index (physical well-being/functional well-being/additional concerns);

‡ FACT-G = General assessment (physical well-being/social and family well-being/emotional well-being/functional well-being)

The means of quality of life of the allogeneic group were lower than those of the autologous group in all domains, showing a significant difference in relation to additional concerns (p=0.01) and in the treatment evaluation index (p=0.04).

When the general assessment score (FACT-G) was correlated with each domain that makes up the FACT-BMT instrument, it was observed that all domains had a statistically significant correlation with the general QoL ( Table 2). This result indicates the existence of an interrelationship between the domains with general quality of life, and impairment of physical, social and emotional aspects in the post-transplant period, during the COVID-19 pandemic. 

**Table 2 -:** Correlations between the general assessment (FACT-G)* and the domains of the Functional Assessment of Cancer Therapy Bone Marrow Transplantation (FACT-BMT)† of patients undergoing autologous and allogeneic transplantation (n=35). Curitiba, PR, Brazil, 2021-2022

	Spearman	*P*
FACT-G* x Physical well-being	0.841	0.000
FACT-G* x Social well-being	0.436	0.008
FACT-G* x Emotional well-being	0.706	0.000
FACT-G* x Functional well-being	0.744	0.000
FACT-G* x Additional concerns	0.690	0.000

* FACT-G = General assessment (physical well-being/social and family well-being/emotional well-being/functional well-being);

† FACT-BMT = Functional Assessment of Cancer Therapy Bone Marrow Transplantation

With regard to financial toxicity, the participants had an average score of 22.11/44, indicating a mild impact and a minimum standard deviation of 1 and a maximum of 37, revealing a great variability of responses ( Table 3). 

**Table 3 -:** Description of the minimum, maximum and average values of the COST* score by type of transplant (n=35). Curitiba, PR, Brazil, 2021-2022

		COST*				
HSCT†	Mean	N	SD‡	Minimum	Maximum	P
Autologous	27.50	6	7.69	15	37	
Allogenic	21.00	29	7.91	1	31	
Total	22.11	35	8.15	1	37	0.084

* COST = COmprehensive Score for Financial Toxicity ;

† HSCT = Hematopoietic stem cell transplantation;

‡ SD = Standard deviation

**Figure 1 -:**
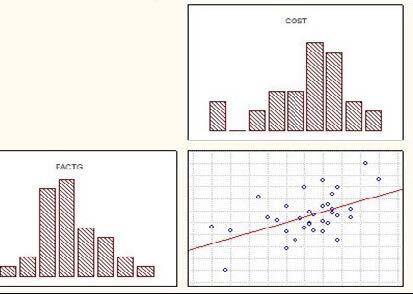
Correlation between general assessment (FACT-G*) and financial toxicity (COST†). Curitiba, PR, Brazil, 2021-2022

When correlating the general assessment (FACT-G) with financial toxicity (COST), a correlation between both is observed, although not significant (p=0.051). It can be seen, in Figure 1 ^,^ that the relationship between them is direct, that is, when the general assessment score increases (FACT-G), the impact of financial toxicity is lower, since the COST score also increases. 

*FACT-G = Functional Assessment of Cancer Therapy General; †COST = COmprehensive Score for Financial Toxicity

## Discussion

HSCT is a long and complex treatment that impacts on the quality of life of the transplant recipient and their families. However, in the scenario of the COVID-19 pandemic, patients, in addition to being faced with the aggressive treatment of the underlying disease, corresponded to a vulnerable group with a higher risk of death. Among the factors that can influence the decline in QoL and the financial toxicity of transplant patients, withdrawal from professional activities deserves to be highlighted, especially when the population is young adults, as in the present study.

The diagnosis of leukemia, more frequent in this study, can often increase anxiety in transplant patients. This type of hematologic cancer causes impaired bone marrow cell production and maturity, which is associated with profound and prolonged immunosuppression, increasing susceptibility to infections and the risk of a severe course of COVID-19 ^(^ 24 ^-^ 25 ^)^. A cohort of 536 cases of symptomatic COVID-19 infection and hematologic cancer observed that 198 (37%) participants died and it was concluded that patients with hematologic malignancies and COVID-19 infection are at high risk of mortality ^(^ 25 ^)^. 

The predominant type of transplant in the present study was allogeneic. The high rate of this modality may be related to the location where the transplant was carried out, as it is a reference center in Latin America, which stands out for the higher number of allogeneic HSCT, differing from the HSCT performed in Brazil in the last ten years, where the number of autologous transplants was annually higher than the other ^(^ 26 ^)^. A cohort study carried out during the pandemic with stem cell transplant recipients showed that the factors associated with a higher risk of mortality after the development of COVID-19 are: 1) male (HR 3.53; 95%CI; 1.44-8.67; P= 0.006) and 2) having undergone allogeneic HSCT in the last 12 months (HR 2.67; 95% CI, 1.33-5.36; P= 0.005) ^(^ 27 ^)^. Both studied variables were the most frequent in the center where the study was carried out. 

Autologous and allogeneic HSCT presents several symptoms related to the toxicity of chemotherapy and radiotherapy ^(^ 28 ^)^. However, in addition to these events inherent to HSCT, during the pandemic, patients had an increased risk of complications associated with COVID-19, which led to impaired QoL ^(^ 29 ^-^ 30 ^)^. This can be observed when analyzing the results of studies carried out in the same institution, with the same instrument, outside the context of the pandemic. The averages of the quality of life domains were higher than those of the present study ^(^ 31 ^-^ 32 ^)^. 

In the general assessment (FACT-G), despite clinical differences and treatment time, patients undergoing autologous and allogeneic HSCT showed similar alterations. When comparing the physical well-being, functional well-being, social and emotional well-being measured by the FACT-BMT, it is observed that the means of the allogeneic group are relatively lower than the autologous ones, with a statistical difference in relation to additional concerns and in the treatment evaluation index. This result converges with a study that analyzed the quality of life in autologous and allogeneic transplant patients and highlighted that there are no statistically significant differences in quality of life between the transplant modalities ^(^ 31 ^)^. However, it is noteworthy that the development of allogeneic HSCT depends on the effort to find a compatible donor, in the management of complications such as graft-versus-host disease (GVHD), which implies changes in the domains of QoL ^(^ 33 ^)^. 

Regarding the domains social well-being and emotional well-being, both statistically significant with the general assessment, it can be considered that they were possibly affected by the need to intensify the physical-social distance. It is common for the transplant recipient to be isolated due to neutropenia, however, with the measure of social isolation to reduce and limit the generalized spread of COVID-19, in many cases the visit even by family members was restrictive, enhancing the impairment of the QoL of these patients.

In an Austrian study ^(^ 34 ^)^, it was found that approximately half of cancer patients undergoing hospital treatment reported limitations in their daily activities due to restrictions of the COVID-19 pandemic, causing damage to social well-being. Among the most cited problems were being fired from work, organizing childcare at home, and loneliness due to lack of contact with family and friends. 

A study conducted in Turkey identified increased anxiety during the COVID-19 pandemic in patients undergoing HSCT. The reported feelings were related to uncertainties about the future, concerns about the results of the treatment and possible worsening of the disease ^(^ 14 ^)^. Increased distress should also be taken into account, as patients had their treatment postponed, canceled or changed, their appointments or medical examinations rescheduled, changing their therapeutic course ^(^ 9 ^)^. Emotional changes are problems reported by cancer patients and have a negative impact on therapy and QoL ^(^ 35 ^)^. 

One study ^(^ 36 ^)^ carried out in Poland analyzed the QoL of a population with cancer during the new coronavirus pandemic and compared the results obtained with a sample of cancer patients in a non-pandemic situation. In the correlation, the group of patients evaluated during the pandemic had a significantly lower average in the performance of the social function. This demonstrates that the pandemic had an impact on issues related to social relationships and work activities, impacting on QoL and being able to generate or intensify financial toxicity in transplant patients. Studies indicate that issues related to financial toxicity, such as concerns about returning to work and financial difficulties, have an impact on the physical and psychological well-being of patients in the post-HSCT period ^(^ 18 ^-^ 19 ^)^ and can influence the continuity and adherence to treatment ^(^ 20 ^)^. 

The review study ^(^ 37 ^)^ which aimed to identify publications on cancer, financial toxicity, and economic challenges in the context of COVID-19, resulted in increased treatment costs, unemployment, diminished quality of life, impaired mental health, and financial toxicity associated with depressed mood. In the same direction, an editorial ^(^ 29 ^)^ reported the reality of Ghana during the COVID-19 pandemic and how cancer patients were affected, pointing to increased psychological suffering, difficulties in accessing health services, rising prices and impaired quality of life. That is, the scenario of the COVID-19 pandemic exacerbated feelings, concerns and conditions that cause damage in several domains involving QoL. 

The average financial toxicity score of the sample in this study indicated, according to the classification of a Japanese study ^(^ 38 ^)^, slight impact, however, it pointed to the presence of the aforementioned toxicity that can be experienced in different ways, depending on the therapy required, the patient’s socioeconomic status, reserves and assets, the need for travel and accommodation to conduct the treatment. A similar result was obtained by the study ^(^ 39 ^)^ carried out in the United States of America, which measured financial toxicity and its effects in 111 patients undergoing treatment for multiple myeloma; of these, 59% reported that treatment costs were higher than expected. The same study emphasized that patients with COST scores below the median, that is, with greater financial toxicity, were the ones who most reported a reduction in spending on basic goods, as well as using savings reserves, borrowing and delaying the start of treatment. 

The correlation between financial toxicity and the general evaluation was statistically significant, suggesting that the financial difficulty presented by the patients is associated with a decrease in the general QoL. The multicenter study carried out in Hong Kong with 640 cancer patients, correlating COST and FACT-G results, identified that patients who had greater financial toxicity were young, with reduced working hours or unemployed and with limited resources to deal with their financial difficulties associated with cancer ^(^ 40 ^)^. Furthermore, financial difficulty triggers changes in lifestyle, which consequently leads to psychological overload and impaired quality of life ^(^ 41 ^)^. 

Financial toxicity has been associated with clinically relevant outcomes such as worse physical and mental health and QoL ^(^ 42 ^)^. In this regard, an American study ^(^ 43 ^)^, who investigated financial toxicity and health-related quality of life in a cohort of cancer survivors, observed that greater financial toxicity was associated with components such as anxiety, physical functioning and social functioning, elements that make up the QoL construct. 

The assessment of QoL is an important indicator for monitoring the patient’s progress and the effectiveness of the interventions carried out. Through it, the specific problems and needs of each patient are identified, promoting individualized and effective care. The results presented for now correspond to the starting point for a new phenomenon to be observed in the context of HSCT.

Thus, the contributions of this study, in addition to the uniqueness of the topic in the Brazilian scenario, are related to: professional practice, in the sense of expanding knowledge about the presence of financial toxicity and its consequences; the research context, by revealing the presence of a new toxicity among transplant recipients and exposing the subject so that other services conduct similar or more in-depth research; to teaching, by offering material on a topic that is current and relevant to the science of health and nursing.

In this research, the small number of participants was a limiting factor. This is possibly due to the reduced number of beds available for HSCT in the hospital where the study was carried out, due to the institutional changes brought about by the pandemic, the difficulty in finding a compatible donor for allogeneic HSCT, as well as therapy with a prolonged period of hospitalization until the reconstitution of hematopoiesis.

## Conclusion

The COVID-19 pandemic had a negative impact on the quality of life of patients undergoing HSCT and had a slight impact on financial toxicity scores. The correlation between QoL and financial toxicity showed that when QoL increases, financial toxicity is lower. Considering the complexity of the transplanted patient in the pandemic context, the understanding that in addition to physical toxicities there are others that can even cause them is of fundamental importance in the elaboration of care actions.

The professionals who work with the transplanted patient, especially the nurse, and the nursing team need to know the domains that change and affect the lives of these patients, to enhance the individualized care plan and the planning of care based on joint work, in the partnership and in the continuous exchange of information with the multidisciplinary team.
